# Sintering Behavior of Bi-Material Micro-Component of 17-4PH Stainless Steel and Yttria-Stabilized Zirconia Produced by Two-Component Micro-Powder Injection Molding Process

**DOI:** 10.3390/ma15062059

**Published:** 2022-03-10

**Authors:** Al Basir, Abu Bakar Sulong, Nashrah Hani Jamadon, Norhamidi Muhamad

**Affiliations:** Department of Mechanical and Manufacturing Engineering, Faculty of Engineering and Built Environment, Universiti Kebangsaan Malaysia, Bangi 43600, Selangor, Malaysia; al.basir005@yahoo.com (A.B.); nashrahhani@ukm.edu.my (N.H.J.); norhamidi@ukm.edu.my (N.M.)

**Keywords:** two-component micro-powder injection molding, 17-4PH/zirconia micro-sized components, sintering, physical properties, mechanical properties

## Abstract

In this research, we investigated the influence of the sintering temperature on the physical and mechanical properties of micro-sized bi-material components of 17-4PH stainless steel and 3 mol% yttria-stabilized zirconia fabricated using a two-component micro-powder injection molding (2C-μPIM) process. First, 17-4PH and zirconia powders were separately mixed with binders to obtain feedstocks, which were then injection-molded into the dumbbell shape, followed by the binder extraction process. Subsequently, the debound micro-specimens were subjected to sintering between 1250 °C and 1350 °C for 3 h. Per the observations of the microstructures using scanning electron microscopy (SEM), a strong bond between metal and ceramic in micro-sized 17-4PH/zirconia components was formed when the sintering temperature exceeded 1300 °C. The maximum relative density of 99% was achieved when the bi-material micro-part was sintered at 1350 °C. The linear shrinkage increased from 9.6% to 17.4% when the sintering temperature was increased from 1250 °C to 1350 °C. The highest hardness value of 1439.6 HV was achieved at 1350 °C along the bi-material bonding region. Moreover, a maximum tensile strength of 13.7 MPa was obtained at 1350 °C.

## 1. Introduction

The influence of micro-systems technology has increased over the past few decades. The world market has emphasized producing downsized products [1,2,3]. The micro-powder injection molding (μPIM) process, a transfiguration of the powder injection molding (PIM) process, is a commercially viable approach to fabricate metal and ceramic-based micro-sized components [4,5,6,7]. The two-component micro-powder injection molding (2C-μPIM) process modifies the μPIM process to join two dissimilar materials at the micro-scale. The fabricated micro-specimens attain several characteristics and functionalities [8,9,10]. The cost-effective production advantages and opportunities to employ a broad range of materials have established the 2C-μPIM as a remarkable manufacturing process in modern times. Piotter et al. [11] reported an industrially utilizable heating element produced based on the 2C-μPIM technique. The 2C-μPIM-processed magnetic and nonmagnetic bi-metal components were fabricated by Imgrund et al. [10] to study their viability in various micro-applications. Ruh et al. [8] produced a shaft-to-collar connection employing 2C-μPIM to validate the feasibility of their selected materials. For long-term sustainability in the global market, it is critical that the 2C-μPIM technology exhibits superior productivity and adequacy to produce non-defective components.

Analogous to PIM and µPIM, the four principal stages of the 2C-µPIM technique are mixing, injection molding, debinding, and sintering. The 2C-µPIM procedure usually starts with the mixing process, where two different types of homogeneous feedstocks are prepared by mixing metal or ceramic powders separately with binders. The rheological analysis is performed on the feedstocks to determine flow behavior to prevent the development of defects in components during the injection molding operation. After injection molding, the obtained bi-material micro-parts are commonly known as green parts, and the sequential mechanism is often used to achieve such parts [12]. The binder extraction process is performed right after the micro-injection molding operation, a significant stage in the 2C-µPIM process. This stage has a high defect formation probability that must be prevented to produce quality parts after sintering [13,14,15]. The most effective debinding technique is to comprehensively perform solvent and thermal debinding [16,17]. While the solvent debinding process facilitates the elimination of the soluble binder in the bi-material micro-part, the key purpose of the thermal debinding process is to remove the insoluble binder [18]. Sintering is the concluding phase of the 2C-µPIM, where the debound two-component micro-sized components are sintered to achieve desired physical and mechanical properties.

Furthermore, 17-4PH stainless steel and 3 mol% yttria-stabilized zirconia are renowned materials in engineering. While 17-4PH is associated with excellent corrosion resistance and mechanical strength, zirconia is well-known for its fracture toughness, flexural strength, excellent wear resistance, good biocompatibility, and high-temperature stability [18,19,20]. The 2C-µPIM-processed bi-material micro-sized components of 17-4PH and zirconia can be used in many engineering applications, such as medical implants, automobile engines, and gas turbines, where metal–ceramic combinations are often used to achieve diverse functionalities at lower cost. The sintering of 17-4PH/zirconia micro-sized components produced through 2C-µPIM is complicated due to the mismatched interface strain. Sintering temperature significantly affects specimens’ physical and mechanical properties. Currently, there are insufficient data depicting the sintering behavior of 17-4PH/zirconia micro-parts. Hence, the current study assesses the challenges of fabricating bi-material micro-parts of 17-4PH and zirconia through the 2C-µPIM technique. The influence of the sintering temperature on the physical and mechanical properties of the micro-specimens is investigated.

## 2. Materials and Methods

**Figure 1:**
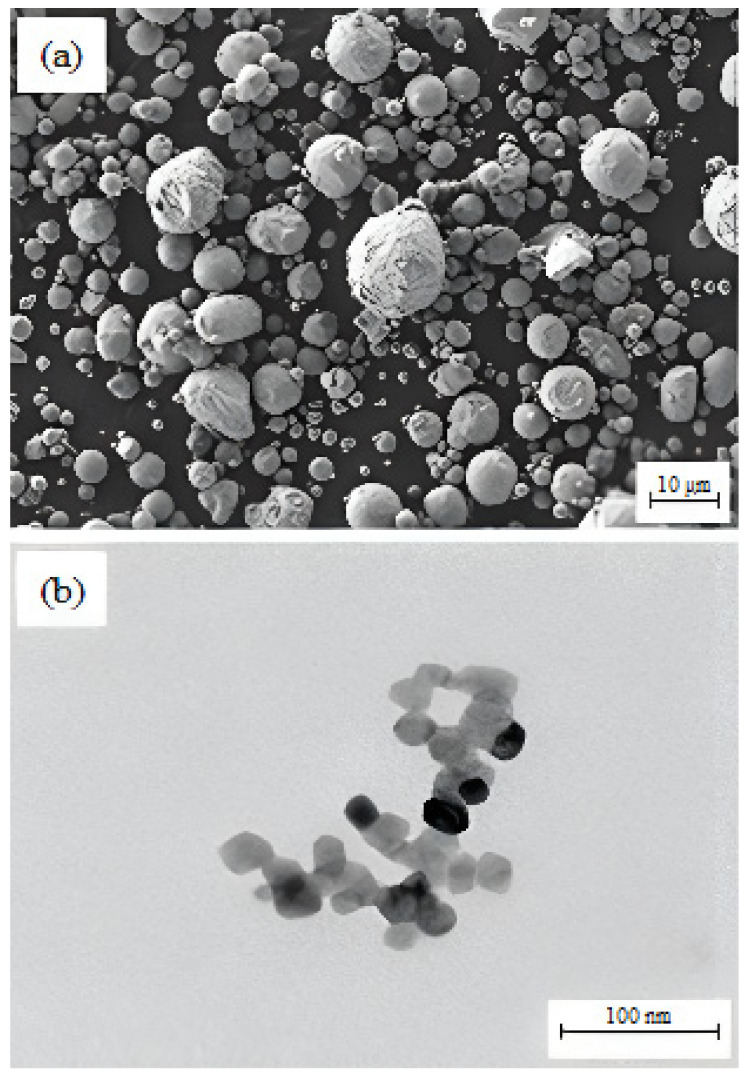
Morphologies of the powders: (**a**) FESEM image of 17-4PH stainless steel particles; (**b**) TEM image of zirconia particles.

The materials used in the current research were 17-4PH stainless steel powder (Sandvik Materials Technology, Sandviken, Sweden) and 3 mol% yttria-stabilized zirconia powder (Inframat Advanced Materials, LLC, Manchester, CT, USA), while the mean particle sizes were 7.5 µm and 172.4 nm, respectively. A field emission scanning electron microscope (FESEM) (Zeiss Merlin Compact, Jena, Germany) and transmission electron microscope (TEM) (Talos L120C, Waltham, MA, USA) were employed to inspect the morphological characteristics of the powders, as displayed in Figure 1. The AccuPyc II 1340 Gas Displacement Pycnometry System (Micromeritics Instrument Corporation, Norcross, GA, USA) was utilized to evaluate the pycnometer densities of 17-4PH and zirconia powders. In this study, the binder system comprised 60 wt.% palm stearin and 40 wt.% LDPE. The palm stearin binder was a major ingredient, having a density of 0.891 g/cm^3^; it was purchased from Sime Darby Kempas Sdn. Bhd. However, Polyolefin Company (Singapore) Pte Ltd. supplied the LDPE binder with a density of 0.91 g/cm^3^, which appeared as the backbone polymer for the green component. Palm stearin was chosen to upgrade the flow properties because it serves as a lubricant and surfactant concurrently. [21]. However, the LDPE was selected to increase the strength of the green part [22]. The different features of the binder components are listed in Table 1.

**Figure 2:**
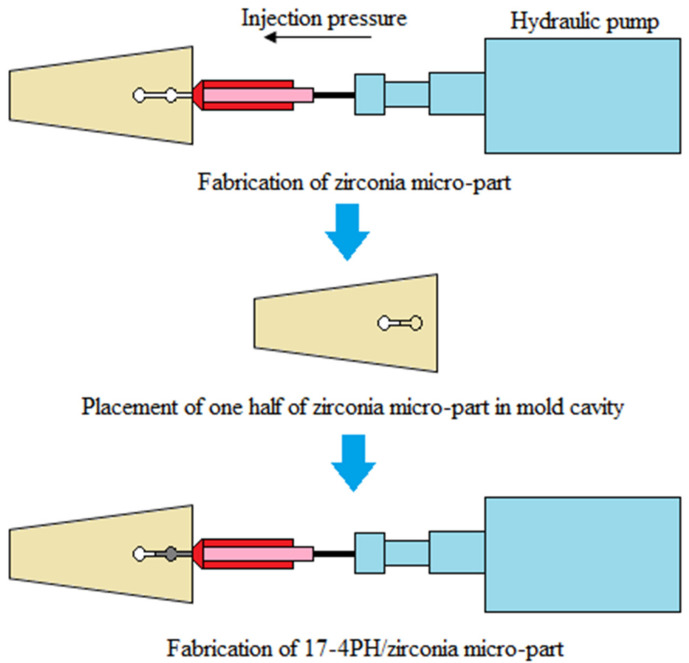
Schematic of the processing method used to produce the green micro-sized 17-4PH/zirconia component, reused with permission from Elsevier [18].

**Figure 3:**
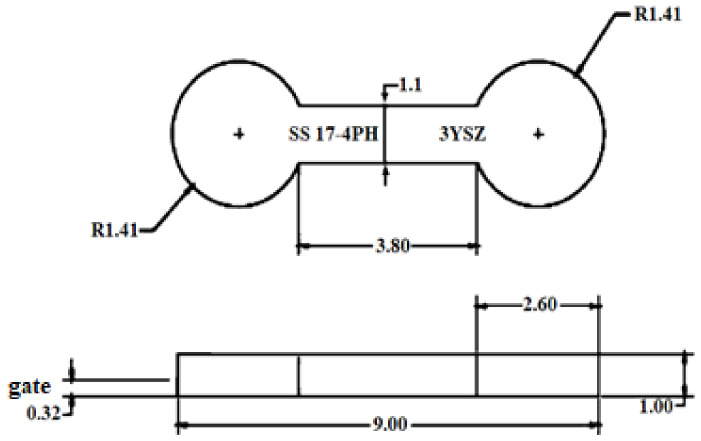
Schematic of the 17-4PH/zirconia micro-sized component (all dimensions are in mm), reused with permission from Elsevier [18].

The 17-4PH and zirconia feedstocks were produced by mixing 69 vol% of 17-4PH and 44 vol% of zirconia powders separately with the binder system composed of palm stearin and LDPE in a Brabender mixer (W50 EHT, Brabender GmbH & Co. KG, Duisburg, Germany). The blade rotation speed was kept at 25 rpm for 45 min at a mixing temperature of 150 °C [12]. Based on Table 1, the mixing temperature exceeded the melting temperature but was lower than the binder decomposition temperatures. Temperature control helped prevent binder degradation during mixing [23]. A DSM Xplore table-top injection molding machine (Xplore Instruments BV, Sittard, The Netherlands) was employed to process the 17-4PH and zirconia feedstocks to fabricate defect-free green micro-sized bi-material components using a sequential mechanism. The processing method schematic is demonstrated in Figure 2. As seen in Figure 2, zirconia feedstock was injected into the mold cavity. The single green zirconia micro-component was then demolded and cut in half before placing it in the mold cavity again. Subsequently, the injection of the 17-4PH feedstock was conducted over the zirconia feedstock, causing the two materials to fuse. The green 17-4PH/zirconia micro-specimens produced based on the previous study parameters [12,18] are displayed in Table 2. The dimensions of the specimen are schematically presented in Figure 3.

Under the formerly investigated parameters, the solvent debinding process was performed using an MMM VentiCell 111 oven (MMM Medcenter Einrichtungen GmbH, Munich, Germany) [18]. To eliminate the palm stearin binder, the green 17-4PH/zirconia micro-sized components were immersed in acetone at 70 °C for 40 min. After solvent debinding, the thermal debinding process was applied on solvent-debound bi-material micro-parts to eliminate the insoluble LDPE and remaining palm stearin binders. Using the tube furnace (HTF-15/200-60), thermal debinding and sintering were conducted successively in an argon atmosphere. The thermal debinding process was composed of two phases. In the first phase, solvent-debound 17-4PH/zirconia micro-sized components were heated from room temperature to 150 °C at a heating rate of 0.1 °C/min. Subsequently, the temperature and heating rates were increased to 550 °C and 0.25 °C/min and a 2 h dwelling period in the second phase.

During sintering, the thermal-debound 17-4PH/zirconia micro-sized components were heated from 550 °C to a 1250–1350 °C temperature range at a 10 °C/min heating rate for 3 h. In selecting sintering temperatures, the previous research by Emeka et al. [24] on the fabrication of bi-material macro-sized components of 17-4PH and zirconia using a two-component powder injection molding (2C-PIM) process was considered. The 17-4PH/zirconia micro-sized components produced using the tube furnace were cooled in three phases. Firstly, the sintered samples were cooled down to 850 °C at a cooling rate of 1 °C/min. Secondly, the cooling was performed at a rate of 0.6 °C/min to reduce the temperature from 850 °C to 450 °C. Finally, the samples were cooled to room temperature from 450 °C at a cooling rate of 0.3 °C/min. The density of the sintered specimens was measured based on MPIF standard 42 using the Archimedes method. The determination of the shrinkage percentage of the samples was carried out by measuring the changes in length between injection-molded green samples and sintered samples following MPIF standard 44. The polished surfaces of the 17-4PH/zirconia micro-sized components were observed using scanning electron microscopy (SEM) (Hitachi SU1510, Schaumburg, IL, USA). The variations in each constituent element present at the interface of bi-material micro-parts were assessed based on energy-dispersive X-ray spectroscopy (EDS) mapping. Pore band formation at the interface of the components was evaluated using optical microscopy (Olympus, BX51M, Tokyo, Japan). The hardness values of the joining region and the 17-4PH and zirconia portions of the bi-materials were measured using a micro-Vickers machine of the HWMMT-X series made by Highwood (Osaka, Japan) following MPIF standard 51. In the case of 17-4PH and zirconia portions, hardness values at three different locations at a distance of 1 mm were taken. During the hardness measurement, a 50 g load was applied for the 1250 °C sintered sample for 15 s, 100 g load was applied for the 1300 °C sintered samples, while 100 g was used for 1350 °C sintered samples using the same period. Finally, the tensile strengths of the two-component micro-specimens were measured following MPIF standard 10 using a universal testing machine (model 5566, INSTRON, Norwood, MA, USA) at a 0.1 mm/min rate with a 1 KN load cell.

## 3. Results and Discussion

**Figure 4:**
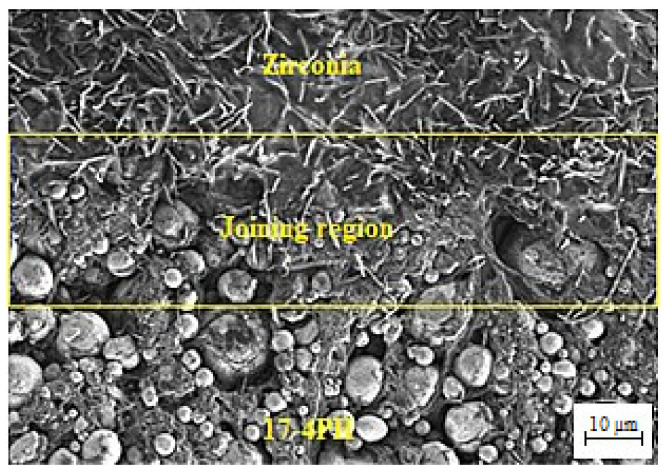
FESEM image of the green 17-4PH/zirconia micro-specimen.

**Figure 5:**
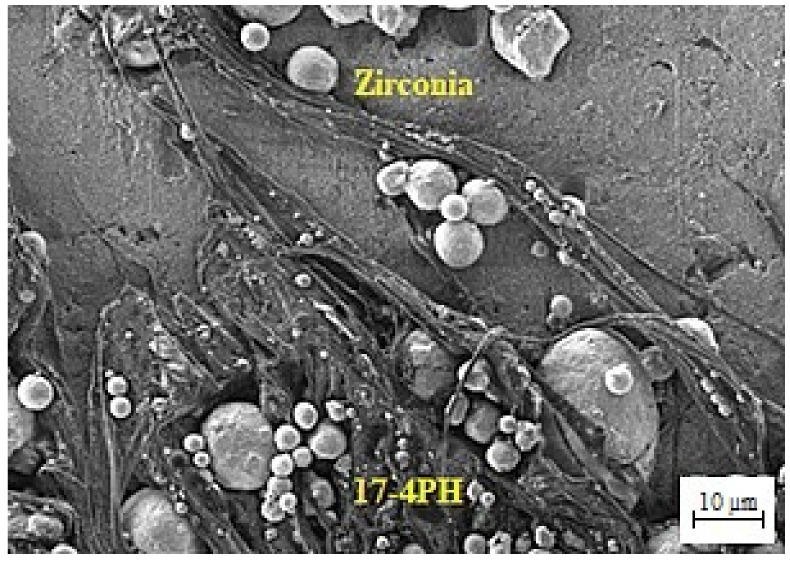
FESEM image of the solvent-debound 17-4PH/zirconia micro-specimen.

**Figure 6:**
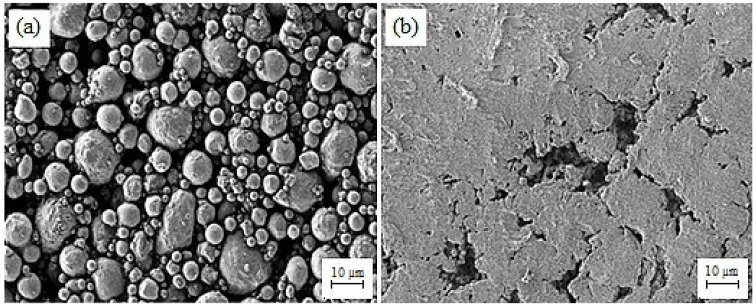
FESEM images of the (**a**) 17-4PH and (**b**) zirconia portions of the micro-sized 17-4PH/zirconia component after thermal debinding.

The prepared feedstock’s flow behavior was considered a significant factor in determining the characteristics of the powder–binder mixture prepared for injection. Different qualities, such as elevated strength and low viscosity, are required to achieve an excellent feedstock. Our previous research demonstrated that the prepared feedstocks of 17-4PH and zirconia with solid loadings of 69 and 44 vol.%, respectively, displayed pseudoplastic behavior or shear thinning at 180 °C, with flow behavior indices of 0.457 and 0.467, respectively [12]. Pseudoplastic behavior typically aided in precise cavity filling on the micro-mold without creating imperfections in micro-sized 17-4PH/zirconia specimens during the 2C-μPIM process. The injection molding results revealed that a mold temperature of 65 °C was employed to fabricate defect-free green bi-material micro-components of 17-4PH and zirconia. Foudzi et al. [25], who fabricated micro-sized zirconia components based on the μPIM process, employed a similar mold temperature during the injection molding stage. Fayyaz et al. [26] produced micro-sized injection-molded specimens of cemented carbide and reported that the mold cavity temperature must be close to the feedstock temperature to ensure that the cavity is filled effectively. The mold temperature is raised to compensate for heat loss throughout the micro-injection molding [27]. The FESEM image of the micro-sized green 17-4PH/zirconia specimen is shown in Figure 4. As depicted in Figure 4, the powder particles of 17-4PH and zirconia were sufficiently covered with the binder system composed of palm stearin and LDPE in the interlocked bi-material specimen. It ultimately enhanced the strength to validate the attributes of the micro-sized specimen in the successive stages. The green micro-sized bi-material specimen structure after solvent debinding is demonstrated in Figure 5. The palm stearin soluble binder was eliminated satisfactorily from the 17-4PH/zirconia micro-part. An open-pore structure facilitated the removal of the insoluble LDPE binder during the thermal debinding stage [28]. The FESEM images of the 17-4PH and zirconia portions of the bi-materials after thermal debinding are shown in Figure 6, confirming the near-complete removal of the binder system.

Determining relative density is considered a pertinent approach to evaluate the sintering process. Usually, an enlargement of the grain size along with a reduction in pore size occurs with an increase in sintering temperature. Due to the molecular diffusion process and grain growth densification, the extermination of pores takes place during the sintering process [29,30,31]. The relative density values of bi-material micro-parts of 17-4PH and zirconia sintered at 1250 °C, 1300 °C, and 1350 °C in an argon atmosphere with a holding period of 3 h are displayed in Table 3. As specified in Table 3, the relative density increased substantially from 94.6% to 98.5% when the sintering temperature was increased from 1250 °C to 1300 °C. The further temperature increase to 1350 °C resulted in the highest relative density of 99%. Typically, PIM-based components should have more than 95% relative density [32]. Considering this point of view, it is recommended to use a sintering temperatures of 1250 °C or higher during the fabrication of the 17-4PH/zirconia micro-sized components so that the samples can reach near-full density.

**Figure 7:**
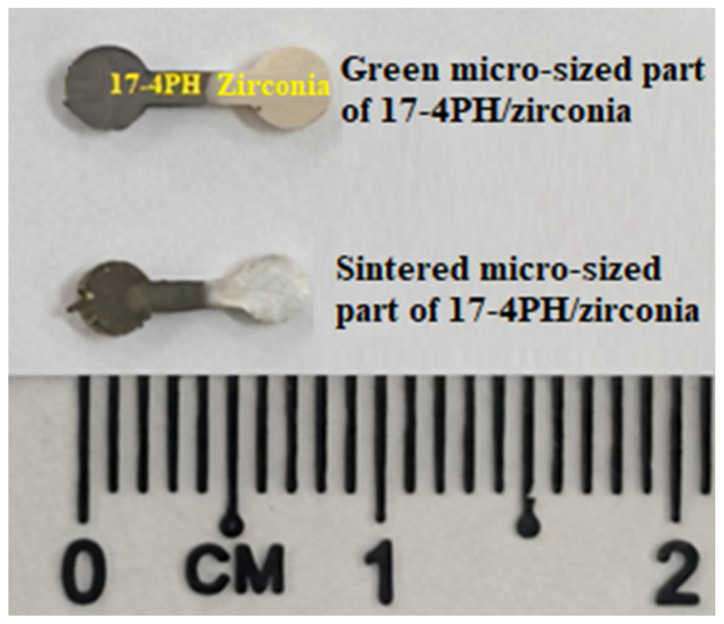
Photograph of green part and debound part sintered at 1350 °C.

**Figure 8:**
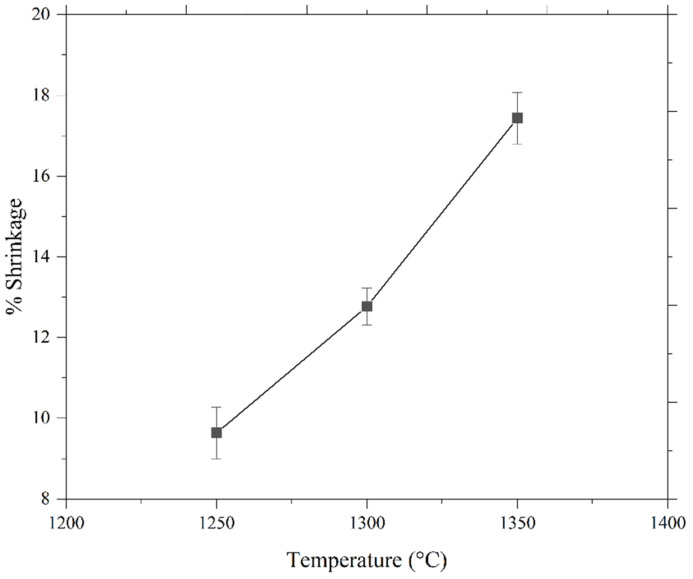
Shrinkage occurred in bi-materials at different temperatures with a dwelling period of 3 h.

Shrinkage occurs in single-material and bi-material micro-sized components, usually during the sintering stage [10,25,33]. Figure 7 exhibits the micro-injection-molded 17-4PH/zirconia component that experienced shrinkage when sintered at 1350 °C with a holding period of 3 h in an argon environment after solvent and thermal debinding. The linear shrinkage percentages of 17-4PH/zirconia micro-sized components at three different sintering temperatures of 1250 °C, 1300 °C, and 1350 °C with a dwelling period of 3 h are displayed in Figure 8. As shown in Figure 8, the shrinkage increased in bi-material micro-parts from 9.6% to 17.4% when the sintering temperature was increased from 1250 °C to 1350 °C. This outcome can be attributed to reduced pore spaces within the powder particles. Consequently, the porosity was low at higher temperatures. Usually, 15% to 25% shrinkage is common in micro-injection-molded micro-parts [34,35]. Shrinkage effects in sintered specimens must be considered when designing the mold and manufacturing the tool so that the final products have the desired shape and size [26].

**Figure 9:**
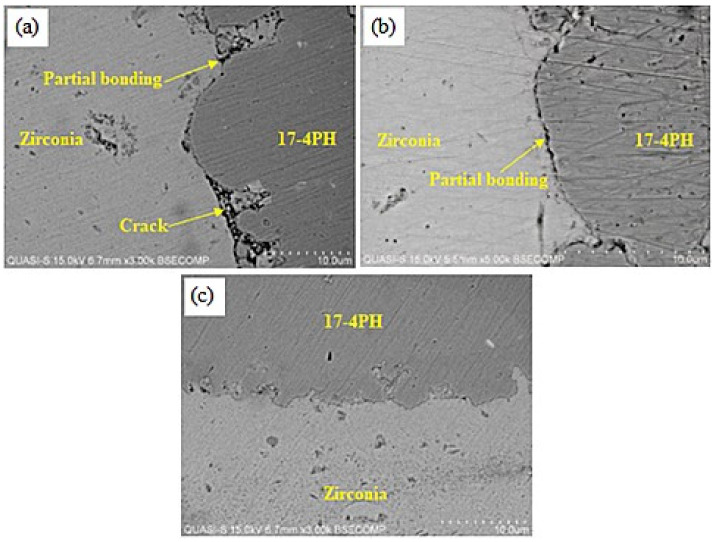
SEM micrographs exhibiting the interfaces of 17-4PH/zirconia micro-sized components sintered for 3 h at various temperatures: (**a**) 1250 °C; (**b**) 1300 °C; (**c**) 1350 °C.

**Figure 10:**
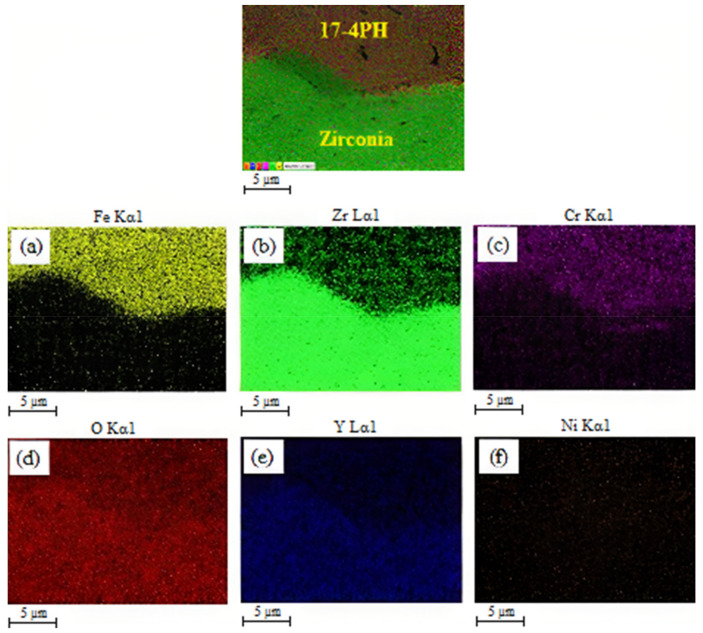
EDS mapping displaying the bonded 17-4PH/zirconia micro-sized component and proportions of (**a**) Fe element; (**b**) Zr element; (**c**) Cr element; (**d**) O element; (**e**) Y element; (**f**) Ni element present across the interface.

**Figure 11:**
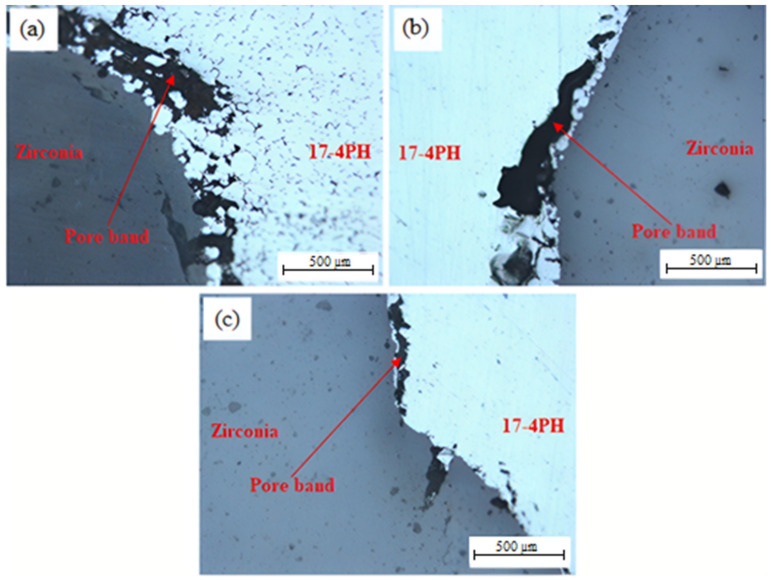
Optical microscopy images displaying the interfaces of 17-4PH/zirconia micro-sized components sintered for 3 h at various temperatures: (**a**) 1250 °C; (**b**) 1300 °C; (**c**) 1350 °C.

The SEM images of the 17-4PH/zirconia micro-parts sintered at 1250 °C, 1300 °C, and 1350 °C with a holding period of 3 h are exhibited in Figure 9. Based on Figure 9a, the partially bonded interface of 17-4PH and zirconia had cracks when the micro-sized specimen was sintered at 1250 °C. During sintering, mismatch strains are produced at the interface as a consequence of the predisposition of different layers of materials to shrinkage at different rates [36]. Such differences in shrinkage rates cause delamination, pore-band formation, and cracking in the bonding region due to bi-axial mismatch stresses [37]. The partial bonding of the specimen was improved when the temperature was enhanced to 1300 °C, as displayed in Figure 9b. As shown in Figure 9c, 17-4PH and zirconia had a strong bond when sintering was performed at 1350 °C; this was attributed to an adequate reduction in mismatch shrinkage. Figure 10 depicts EDS mapping. Iron (Fe), zirconium (Zr), chromium (Cr), oxygen (O), yttrium (Y), and nickel (Ni) were present at the 17-4PH/zirconia interface of the micro-sized component. The inter-diffusion of elements at the interface was caused by their higher affinity toward the oxygen of the zirconia ceramic, which ultimately caused bonding between 17-4PH and zirconia due to oxide layer formation [38]. Figure 11 illustrates the microstructures of the 17-4PH/zirconia micro-sized components sintered at different temperatures with the existence of pore bands. As Ni and Cr possess greater diffusion coefficients in Fe than Zr diffusing from the opposite side, vacancies develop in the vicinity of the 17-4PH stainless steel portion, creating pore bands [39].

**Figure 12:**
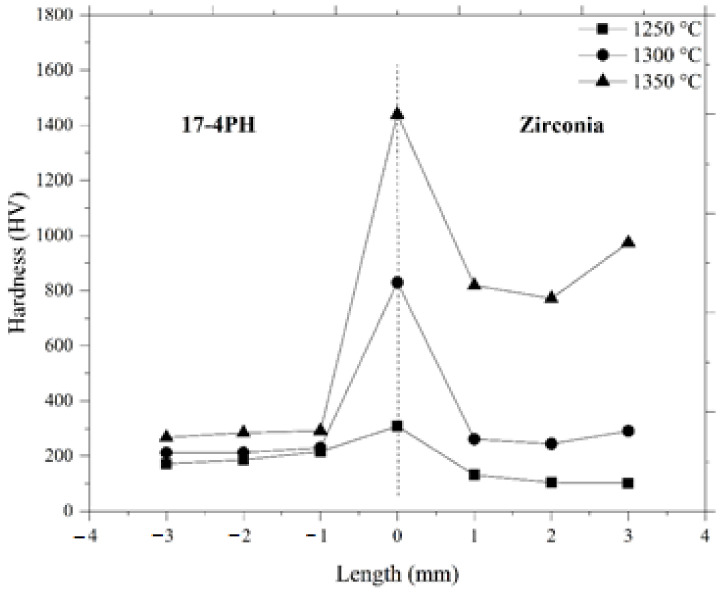
Hardness values of 17-4PH/zirconia micro-parts sintered at different temperatures.

**Figure 13:**
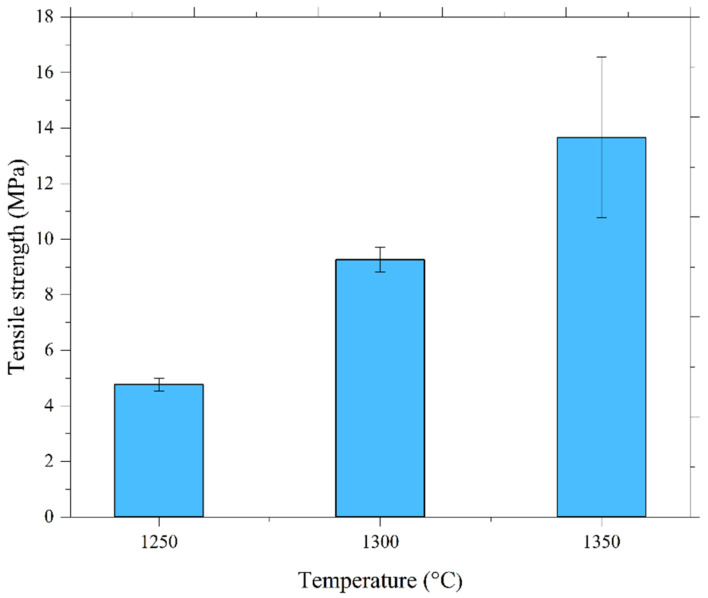
Tensile strength versus sintering temperatures.

Hardness variations in 17-4PH/zirconia micro-sized components at 1250 °C, 1300 °C, and 1350 °C sintering temperatures are displayed in Figure 12. The bonding region of 17-4PH and zirconia materials is represented by a dotted center line in Figure 12. In this study, the hardness along the bonding region increased drastically from 308 HV to 1439.6 HV when the sintering temperature was increased from 1250 °C to 1350 °C. This was attributed to reduced pore band development at higher temperatures. Moreover, as depicted in Figure 12, the hardness values at different locations of the 17-4PH and zirconia portions of the bi-materials increased with sintering temperature. This was attributed to reduced porosity with temperature increases [40]. In this research, we measured the tensile strength of the 17-4PH/zirconia micro-sized components sintered at different temperatures. As exhibited in Figure 13, the 1250 °C sintering temperature yielded a 4.8 MPa tensile strength, which increased to 13.7 MPa when sintered at 1350 °C. Pore band reductions in bi-materials with increasing temperature may have been responsible for this outcome. During this experiment, brittleness-induced fracture occurred expeditiously in the zirconia portions of the 17-4PH/zirconia micro-parts sintered at 1250 °C, 1300 °C, and 1350 °C; however; no failure was observed in the bonding regions of 17-4PH and zirconia materials. It indicated that the zirconia portion was weaker than the 17-4PH portion and bonding region. Compared to the other areas, this can be attributed to the lower strength of the zirconia portion to accommodate the applied load before failure.

## 4. Conclusions

The objective of this research was to investigate the physical and mechanical properties of the sintered metal–ceramic micro-specimens. The sintering process was successfully performed on the solvent- and thermal-debound 17-4PH/zirconia micro-sized components with dimensions of a few millimeters at a temperature range of 1250 °C to 1350 °C for 3 h. More than 98% relative density was achieved at sintering temperatures of 1300 °C or higher. The sintered components had 17.4% more linear shrinkage than the green ones at 1350 °C; lower sintering temperatures significantly reduced the shrinkage. The sintered microstructures showed that 17-4PH stainless steel and zirconia bonding improved gradually in micro-sized bi-materials when the sintering temperature was increased from 1250 °C to 1350 °C. The sintering temperature of 1350 °C was most suitable for 2C-μPIM-processed components because the hardness and tensile strength were optimal at 1439.6 HV and 13.7 MPa, respectively.

## Figures and Tables

**Table 1:** Properties and characteristics of the binders.

Binders	Content (wt%)	Chemical Designation	Melting Temperature (°C)	Range of Decomposition (°C)
Palm stearin	60	CH_3_(CH_2_)_14_COOH	49.8	355.8–465.9
LDPE	40	(C_2_H_4_)*n*	109.2	397.8–501.4

**Table 2:** Micro-injection molding parameters.

Melt Temperature (°C)	Mold Temperature (°C)	Injection Pressure (bar)	Injection Time (s)	Holding Pressure (bar)
180	65	10	7	10

**Table 3:** Relative density of 17-4PH/zirconia micro-sized components sintered at different temperatures for 3 h.

Sintering Temperature (°C)	Relative Density (%)
1250	94.6
1300	98.5
1350	99.0

## Data Availability

Data that supporting the results of this study are available from the first author.
